# Active secretion of CXCL10 and CCL5 from colorectal cancer microenvironments associates with GranzymeB+ CD8+ T-cell infiltration

**DOI:** 10.18632/oncotarget.3205

**Published:** 2014-12-11

**Authors:** Timothy J. Zumwalt, Mildred Arnold, Ajay Goel, C Richard Boland

**Affiliations:** ^1^ Gastrointestinal Cancer Research Laboratory, Baylor Research Institute and Sammons Cancer Center, Baylor University Medical Center, Dallas, Texas, USA; ^2^ Institute of Biomedical Studies, Baylor University, Waco, Texas, USA; ^3^ Baylor Institute for Immunology Research, Dallas, Texas, USA

**Keywords:** Colorectal cancer, Helper T-cell, Cytotoxic T-Lymphocyte, Immune Cell; Chemokine

## Abstract

Transcriptional expression of CXCR3 and CCR5 cognate chemokines correlate with CD8^+^ T-cell infiltration and prolonged survival in colorectal cancer (CRC). These findings were derived mainly from paraffin embedded tissues; thus little is known about the secretion pattern of CD8^+^ T-cell targeting chemokines from CRCs. Therefore, we developed and introduced a novel platform that assesses the immune mediators that are secreted from live excised tissues. Transcriptional profiling and unsupervised hierarchical clustering of 43 CRCs based on expression of genes that represent the adaptive immune response were used to predict tumors that are strong secretors of T-cell targeting chemokines. Secretion of these mediators were corroborated using flow cytometric analysis of T-cell lineage markers: CD4, CD8, IFN-γ, and GzmB. We demonstrate that stronger secretion of CXCL10 (CXCR3 ligand) and CCL5 (CCR5 ligand) and infiltration of GzmB^+^CD8^+^ cytotoxic T-lymphocytes (CTLs) and IFN-γ^+^CD4^+^ helper T-cells can be predicted by transcriptional profiling, and that CRCs with stronger T-cell immunity were proportionally skewed towards early TNM stages and lacked distant organ metastasis. Our study represents the first functional analysis of secreted immune mediators from CRCs beyond immunohistochemistry and real-time PCR, and observed active physiological interactions between the tumor cells and the immune cells in the tumor microenvironment.

## INTRODUCTION

Tumor-targeting T-cells have attracted considerable attention over recent years since first demonstrated as beneficial for rectal cancer patients [1]. The variety of T-cell subsets that can infiltrate tumors confound the relationship between tumor-infiltrating T-cells and clinical outcome, *i.e.* the type and density of tumor infiltrating T-cells varies among colorectal tumors and affects metastasis and disease [2-8]. Therefore, a clearer understanding of the immune mediators that dictate T-cell infiltration into the tumor microenvironment (TME) will allow more targeted approaches be tailored for each patient.

The current perception of T-cell infiltration in colorectal TMEs is mainly derived from transcriptional studies and low dimensional immunohistochemistry (IHC) generated from paraffin embedded tissues, usually in the form of tissue microarrays, which limit each antibody to a small amount of tissue [6-9]. Although these transcriptional studies have identified biomarkers that predict disease progression, they fail to correlate active secretion of immune mediators with functional and living infiltrating T-cells and risk reporting biomarkers that may be phenotypically irrelevant. At the same time, while IHC provides spatial discrimination of T-cell infiltrates, it lacks the ability to multi-dimensionally distinguish T-cell subsets that are functionally active [10]. Therefore studying only fixed cells has created a major knowledge gap that highlights the imperative need to assess secreted mediators from live colorectal tumors and to determine whether transcriptional studies are actually translatable to real phenomena.

CD8^+^ T-cells develop into cytotoxic T-lymphocytes (CTLs) and eliminate neoplastic cells by releasing cytotoxic mediators, such as granzyme B (GzmB) and granulysin (Gnly). Interferon gamma (IFN-γ) recruits and activates immune cells through upregulation of adhesion molecules and transcription of IFN-γ-response genes [11, 12]. Type-1 CD4^+^ helper T-cells (T_H_1 cells) polarize from naïve T-cells upon stimulation by interleukin (IL)-12 and upregulation of T-box 21 (*TBX21*) and mediate CTL proliferation and activation, tumor-rejecting immunity, cellular immune responses, and acute inflammation by secreting IL-2, IFN-γ, and tumor necrosis factor (TNF) [13, 14]. Therefore, expression of type-1 T-cell associated genes *CD8A*, *GZMB*, *TBX21*, *IL12RB1*, *IL12RB2*, *CCR5*, *IFN-γ*, interferon regulatory factor 1 (*IRF1*), and signal transducer and activator of transcription 1 (*STAT1*) and the strength of the type-1 response in the TME inversely correlate with tumor relapse and early signs of metastasis [2, 5, 7, 8].

Chemokines are part of a complex network of inflammatory mediators that dictate the type and density of the T-cell population in sites of inflammation [14]. IFN-γ induces (C-X-C motif) receptor 3 (CXCR3) ligands (CXCL9, CXCL10, and CXCL11) to target and attract CTLs and active T_H_1 cells [15-19]. Additionally, CCL5 is critical for T-cell chemotaxis and infiltration as suggested by its receptor (C-C motif) receptor 5 (CCR5) co-expressing with CXCR3 on CTLs in the invasive margin of colorectal tumors. The expression of both receptors has been linked with CTL infiltration and the absence of metastasis [20-22]. Further transcriptional studies have identified specific chemokines (*CX3CL1*, *CXCL9*, *CXCL10*, *CCL2*, *CCL5*, and *CCL11*) and adhesion molecules such as intercellular adhesion molecule 1 (*ICAM1*) as closely associated with T-cell densities and better survival [9]. Two other ligands of CCR5, CCL3 and CCL4, are also highly transcribed in colorectal cancers (CRCs) [21]. Many of these biomarkers have been suggested to represent the type-1 T-cell response, and supplement TNM staging and prognosis [4, 9, 23].

In this study we introduced a novel method that identified the chemokines that are most likely to attract CD8^+^ T-cells to the tumor by evaluating the magnitude of secretion from live CRC tissues. The analytical power of this method is reflected by three observations: early stage tumors secreted more IFN-γ when compared to late stage tumors, CRCs with increased type-1 T-cell activity strongly secreted both CXCL10 and CCL5, and infiltration of functionally active GzmB^+^ CD8^+^ T-cells positively correlated with both the T-cell targeting chemokines. This novel platform utilizes multiplex immunoassays that can improve disease evaluation, identify the immune mediators that are biologically relevant, and be used to improve development of future immunotherapies in cancer by evaluating a more complete milieu of tumor and immune cells.

## RESULTS

### CRCs demonstrate two major patterns of T-cell related transcriptional expression

**Figure 1:**
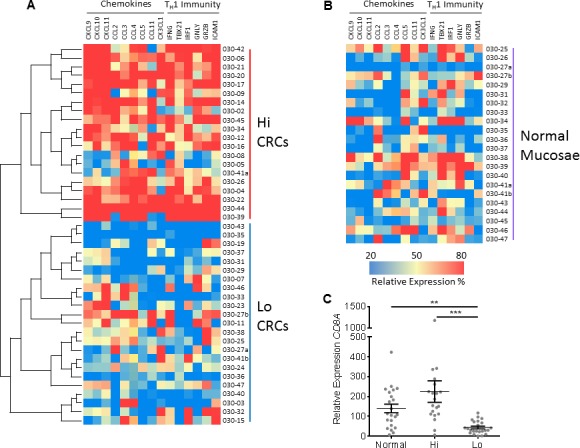
Two major groups of CRCs are identified by transcriptional expression A, Using hierarchal clustering, 43 CRCs were classified into two groups according to transcriptional profiles of genes involved in adaptive immunity (T_H_1) and immune cell chemotaxis. This bifurcation is depicted as a heat map and as a separation of CRCs into either higher (Hi) (n=20) and lower (Lo) (n=23) expressing groups. B, Normal adjacent mucosae (n=22) were included as a comparison, but were not included in the hierarchal clustering. C, Expression of *CD8A* mRNA between normal mucosae and the 2 CRC groups. All genes were normalized to *GAPDH* and Student's *t*-test was performed to determine significance. Error bars represent +/− standard error of the mean (SEM) of grouped tissues. **, *P*<0.01 and ***, *P*<0.001.

The heterogeneity of T-cell infiltration cannot be ignored; proper categorization of CRCs by the magnitude of T-cell activity provides an appropriate scheme for assessing secreted immune mediators [2, 4, 24]. The expression of genes involved in T-cell chemotaxis and T_H_1 immunity were measured via real-time PCR to evaluate the heterogeneity of anti-tumor immune activity across a population of CRCs. Hierarchical clustering was used to bifurcate the population into unique groups. Each tumor was identified as either ‘Hi’ (n=20) or ‘Lo’ (n=23) depending on the group they clustered with (Fig. 1A). Normal adjacent mucosae were included as a comparison (Fig. 1B). The validity of the bifurcation was confirmed by demonstrating higher expression of *CD8A* in the Hi group (Fig. 1C) as well as 13 of the 15 (87%) genes listed in Fig. 1A and B (Supplemental Table 1). This grouping was later used to predict which tumors were more infiltrated with CD8^+^ T-cells and which were stronger secretors of T-cell targeting chemokines.

### T-cell related transcriptional expression predicts CRC progression

**Figure 2:**
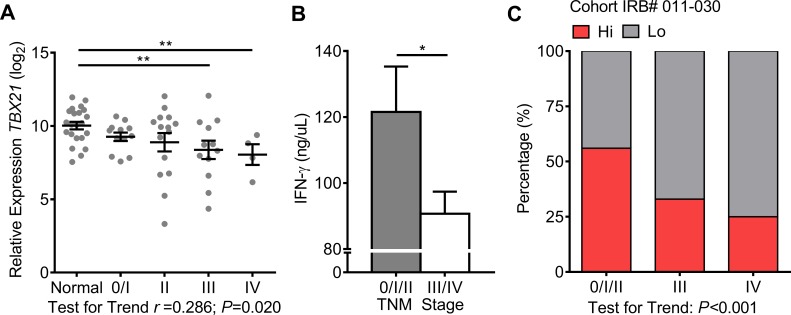
Type-1 T-cell activity decreases with advanced TNM stages A, *TBX21* mRNA expression was measured across normal mucosae and TNM stages. Normal n=22, stage 0/I n=12, II n=15, III n=12, and IV n=4. One-way ANOVA test for linear trend was performed. B, Concentration of secreted IFN-γ was measured from recently resected tissues minced into <1 mm^3^ pieces and placed into culture media for 16 hours. Stage 0/I/II n=18 and stage III/IV n=15. C, The chi-square test for linear trend was performed on CRC groups among TNM stages, stage 0/I/III n=27, III n=12, and IV n=4. Expression values of *TBX21* were log_2_ transformed. Student's *t*-test was performed to determine significance of *TBX21* and IFN-γ. Error bars represent +/− SEM of staged tissues. *, *P*<0.05 and **, *P*<0.01. *P*<0.05 significant for linear trend.

Escape from immune detection leads to tumor metastasis [25]. Therefore, type-1 T-cell activity in colorectal TMEs is expected to decrease as tumors spread and metastasize. TNM stages 0, I, and II are associated with a more favorable prognosis while stages III and IV represent regional lymph node involvement and distant metastasis, respectively [26]. One-way ANOVA test for trend confirmed that *TBX21* decreased with disease progression (*r* = 0.286; *P* = 0.020) (Fig. 2A).

These data impelled the study to examine the secretion of T_H_1 (IFN-γ) and, conversely, T_H_2-associated cytokines (IL-4, IL-5, and IL-13) from live tissues. Normal mucosae and center portions of CRCs were immediately collected after surgery, washed in DL-dithiothreitol (DTT) to remove the mucus layer, minced into small pieces, and cultured in media for 16 hours. Supernatants were then collected, cleared of debris, and analyzed for secreted cytokines using the EMD Millipore's MILLIPLEX Human Cytokine/Chemokine Luminex kit. Due to the small size of the study, data were consolidated by non-metastatic or metastatic stages to reinforce findings. As expected, IFN-γ was secreted more strongly from stage 0/I/II tumors when compare to stage III/IV tumors (Fig. 2B). No difference was detected for secreted IL-4, IL-5, and IL-13 (Supplemental Fig. 1), nor for secreted IFN-γ or *TBX21* expression across degree of depth of invasion (T) (Supplemental Fig. 2). These data confirmed that type-1 T-cell activity in TMEs of CRCs declined with either lymph node or distant organ metastasis.

The chi-square test for linear trend in proportions was used to determine whether the proportion of either tumor group favored early or late TNM stages. This test analyzes categorical data to determine whether an association exists between two separate variables, one variable having two groups (Hi and Lo) and the other having three (TNM stages 0/I/II, III, and IV). The Hi CRCs skewed towards earlier TNM stages (0/I/II) while the Lo CRCs skewed towards later stages (III and IV) (*P*<0.001) (Fig. 2C). These data demonstrate that the Hi group represents CRCs that mainly lack invasion into lymph nodes or metastases to distant organs, while the Lo group represents advanced stage tumors.

### CRCs with decreased type-1 T-cell activity are proportionally skewed towards distant organ metastasis

The evaluation of the anti-tumor immune responses within CRCs is important for patient prognosis, therefore determining the association of type-1 T-cell activity with clinical pathological characteristics was addressed [3, 8, 9, 27]. The small number of tumors collected from cohort of CRC patients enrolled (IRB#011-030) lacked statistical power; therefore this study included a second cohort (n=221) extracted from The Cancer Genome Atlas (TCGA). The results shown are in whole or part based upon data generated by TCGA Research Network [28]. The TCGA cohort was distributed into contingency tables and analyzed using Fisher's exact test to determine whether the Hi and Lo groups had a propensity to distribute by certain clinicopathological characteristics. Fisher's exact test can be employed to analyze categorical data when sample sizes are small. Here, one variable was separated by type-1 T-cell activity (Hi and Lo), and the other variables are listed in Table 1. Patients were separated by the depth of invasion (T) of their tumors into T0/T1/T2 and T3/T4 groups. No differences were determined between the mean ages of Hi and Lo groups of patients (Table 1). The TCGA cohort did not demonstrate a difference in proportion by gender, location of primary tumor, depth of tumor invasion, or lymph node metastasis (N); however it did demonstrate the Lo group to proportionally skew towards distant organ metastasis (M) (*P* = 0.026). These data confirmed that metastatic CRCs can be characterized by relative expression of immune related genes.

**Table:** Clinicopathological characteristics of the TCGA cohort and discrimination of type-1 T-cell genes

	Total	Hi	Lo	Test
Tumors (n)	221 (100%)	110 (100%)	111 (100%)	
Age (y;^$\bar{X}$^ ± SD)	69.42 ± 11.49	70.31 ± 10.97	68.53 ± 11.98	0.251
Gender				
Male	116 (52%)	58 (53%)	58 (52%)	1.000
Female	105 (48%)	52 (47%)	53 (48%)	
Primary tumor				
Colon	156 (71%)	83 (75%)	73 (66%)	0.140
Rectum	65 (29%)	27 (25%)	38 (34%)	
Depth of invasion				
pT0/pT1/pT2	54 (24%)	25 (23%)	29 (26%)	0.639
pT3/pT4	167 (76%)	85 (77%)	82 (74%)	
LN metastasis				
N0	134 (61%)	69 (63%)	65 (59%)	0.583
N1-3	87 (39%)	41 (37%)	46 (41%)	
Organ Metastasis				
M0	185 (84%)	98 (89%)	87 (78%)	**0.026**
M1-2	35 (16%)	11 (10%)	24 (22%)	
MX	1 (<1%)	1 (1%)	0 (0%)	
			***P* <0.05 in bold**	

Tumors were distributed into contingency tables and analyzed using Fisher's exact test to determine whether the Hi and Lo groups distributed by certain clinicopathological characteristics. P<0.05 is significant for linear trend. Abbreviations: T, depth of invasion; N, lymph node metastasis; M, distant organ metastasis.

### CRCs with increased type-1 T-cell activity are more highly infiltrated with functionally active T_H_1 cells and CTLs

**Figure 3:**
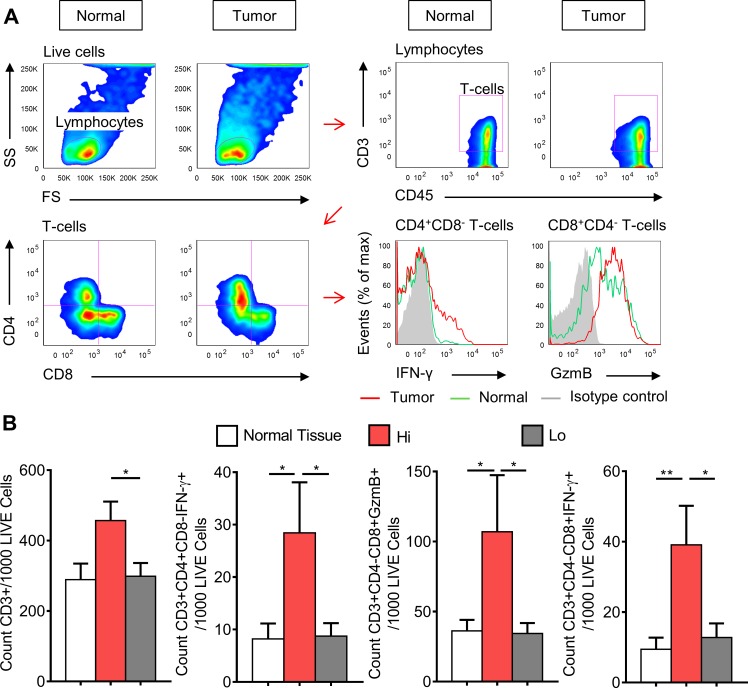
CRCs with high expression of type-1 T-cell genes have higher frequency of infiltrating IFN-γ^+^ helper T-cells and CTLs, and GzmB^+^ CTLs A, Gating strategy used to identify T-cells subsets. Panel A represents one stage I ‘Hi’ CRC and its matched normal mucosa. One stage IV ‘Lo’ CRC is shown in Supplemental Fig. 3. Recently resected normal mucosa and tumor tissue specimens were disaggregated into single cell suspensions, stimulated with PMA and ionomycin, and then stained for seven-color flow cytometric analysis. B, Average counts of total T-cells (CD45^+^CD3^+^), IFN-γ^+^ T_H_1 cells (CD45^+^CD3^+^CD4^+^CD8^−^IFN-γ^+^), IFN-γ^+^ CTLs (CD45^+^CD3^+^CD4^−^CD8^+^IFN-γ^+^) and GzmB^+^ CTLs (CD45^+^CD3^+^CD4^−^CD8^+^GzmB^+^), grouped into normal mucosae (n=14), Hi (n=5), and Lo (n=11) CRCs. Student's *t*-test was performed using total counts per 1000 live cells. Error bars represent +/− SEM of grouped tissues. *, *P*<0.05 and **, *P*<0.01.

Flow cytometry was performed to determine the frequency of functionally active CTLs and T_H_1 cells infiltrating colorectal tumors, and to further validate the immunological characteristics both Hi and Lo groups. Total T-cells were identified from the lymphocyte population as CD45^+^CD3^+^, while functionally active T_H_1 cells were identified as CD4^+^CD8^−^IFN-γ^+^ and functionally active CTLs were identified as CD4^−^CD8^+^GzmB^+^ and CD4^−^CD8^+^ IFN-γ^+^ (Fig. 3A). Total T-cells, IFN-γ^+^ T_H_1 cells, GzmB^+^ CTLs, and IFN-γ^+^ CTLs were increased in Hi CRCs when compared to Lo CRCs; however no difference was detected between normal mucosae and the Lo CRCs for any T-cell subset (Fig. 3B). These data confirmed that CRCs with increased expression of genes involved in T-cell chemotaxis and T_H_1 immunity have a higher frequency of infiltrating and functionally active tumor-targeting T-cells.

### CXCL10 and CCL5 are strongly secreted from CRCs with increased type-1 T-cell activity

**Figure 4:**
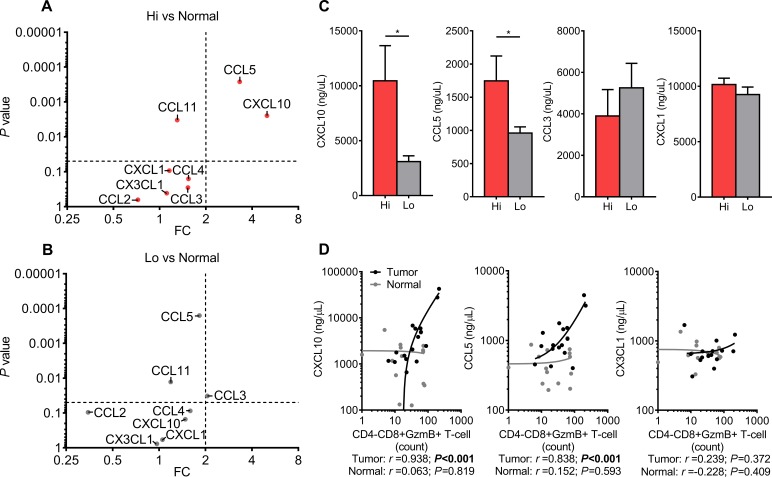
Type-1 T-cell attracting chemokines are strongly secreted from CRCs with increased type-1 T-cell activity A (Hi CRCs vs Normal) and B (Lo CRCs vs Normal), Multiplex Luminex immunoassay of eight chemokines secreted from freshly resected CRCs and normal mucosa. Dot plots of *P* values and fold changes (FCs) between CRC groups and normal mucosa. Dashed lines are thresholds for significant and strong secretion of chemokines (*P*<0.05, and FC>2). Normal n=24, Hi CRCs n=15, and Lo CRCs n=18. C, Comparison of secreted CXCL10, CCL5, CCL3, and CXCL1 between Hi CRCs and Lo. D, Dot plots of chemokine concentrations versus frequency of GzmB^+^ CTLs. Normal n=15 and tumor n=16. Student's *t*-test was performed using concentrations of chemokines. Error bars represent +/− SEM of grouped tissues. *, *P*<0.05. *P*<0.05 is significant for linear trend.

To determine whether T-cell targeting chemokines are actively secreted from CRCs with increased T-cell activity, supernatants from CRCs and normal mucosae were again assayed using the EMD Millipore's MILLIPLEX Human Cytokine/Chemokine Luminex kit [9]. Enough tissue was collected to detect Chemokine concentrations within the optimal ranges of the assay (data not shown); therefore stimulatory agents were not used. Culturing was limited to 16 hours because longer incubations led to signal degradation (data not shown). A fold change (FC) and *P* value for each secreted chemokine was calculated by comparing the Hi and Lo groups to normal mucosae. A FC of >2 and *P* value <0.05 were set as the thresholds for strong secretion. CXCL1 was included as a control because to our knowledge no study has indicated it as strongly expressed with type-1 T-cell activity in CRCs. As expected, strong secretion of both CCL5 and CXCL10 were detected from Hi CRCs when compared to normal mucosae (Fig. 4A). Secretion of both were not increased from Lo CRCs, however CCL3 did meet the criteria as strongly secreted (Fig. 4B). Interestingly, secretion of CCL2, CCL4, CX3CL1, and CCL11 were not increased in either group. A direct comparison of CRC groups showed that CXCL10 and CCL5 secretion are increased in Hi CRCs when compared to Lo CRCs, however CCL3 and CXCL1 were not significantly increased in either group (Fig. 4C). These data demonstrated that CXCL10 and CCL5 are more strongly secreted from CRCs with comparatively stronger type-1 T-cell activity.

Since CCL5 and CXCL10 secretion is enhanced in colorectal tumors with strong type-1 T-cell activity, the frequency of T-cells should correlate with secretion of both chemokines. CTL frequency in tumors and secreted chemokine concentrations were measured earlier via flow cytometry and Luminex. CX3CL1 was included as a negative control because its FC was approximately 1.0 with both CRC groups when compared to normal mucosae. As expected, the frequency of GzmB^+^ CTLs positively correlated with CXCL10 and CCL5 in tumors (*r* = 0.938 and 0.838, respectively; *P*<0.001 for both) but not with CX3CL1, and this pattern was not observed among normal mucosae for any of the three chemokines (Fig. 4D). However, IFN-γ^+^ T_H_1 cells did not appear to follow the same pattern for any cytokine (Supplemental Fig. 4). These data suggest that this novel method of immunoassaying determined that CCL5 and CXCL10 are strongly secreted from colorectal tumors with high infiltration of cytotoxic lymphocytes and that these chemokines are biologically relevant in colorectal tumors.

## DISCUSSION

The prognostic relevance of T-cell biomarkers in human cancer has been the focus of much debate. In general, type-1 T-cell activity is important for prolonged patient survival [8, 27]. Chemokines and adhesion molecules function in biomolecular networks to facilitate T-cell infiltration into sites of inflammation. However the specific chemokines critical for T-cell infiltration into CRCs have not been fully investigated. Our novel method for analyzing secreted immune mediators clarified which biomarkers are critical and relevant for anti-tumor activity. This platform allowed us to quantitate the dynamic range of immunological activity in CRCs beyond transcriptional profiling and low dimensional IHC. To the best of our knowledge, this is the first study to demonstrate the enhanced secretion of CXCL10 and CCL5 from CRCs with strong type-1 T-cell activity. Our data showed that two of the chemokines identified by Mlecnik and colleagues [9] as main contributors of CTL chemoattraction and indicators of prolonged survival are strongly secreted from CRCs with increased type-1 T-cell activity, confirming this observation in living clinical material. However, CCL2, CCL11, and CX3CL1 were not strongly secreted from either CRC group. This could be evidence that increased transcriptional expression of these chemokines is merely an artifact of inflammation that is associated with the adaptive T-cell response. These conclusions could only be derived by developing a novel method for measuring secreted immune mediators from tumors.

Our method was not limited to chemokine analysis. We show that the IFN-γ secretion decreases in CRCs that either metastasized to lymph nodes or distant organs. This indicates that the adaptive T-cell response is inhibited in the 2 metastatic behaviors seen in stages III and IV. These data are consistent with other studies that suggest tumor-targeting T-cell activity is associated with prolonged patient survival and the lack of metastatic disease [2-6, 8].

Our data confirm that the bimodality of gene expression involving T-cell chemotaxis and T_H_1 immunity are key prognostic features of CRC. CRCs with higher expression of these genes were more infiltrated with IFN-γ^+^ T_H_1, IFN-γ^+^ CTLs, and GzmB^+^ CTLs than low-expressing tumors, and were proportionally skewed towards early TNM stages, which suggest that patients with these tumors will survive longer. These observations reflect the major findings pertaining to T-cell infiltration and CRC patient survival [2-8]. As expected, CXCL10 and CCL5 secretion were positively correlated with the frequency of GzmB^+^ CTLs. Interestingly, no correlation was detected between CXCL10 and CCL5 secretion and IFN-γ^+^ T_H_1 cell frequency. This may be due to the low number of IFN-γ^+^ cells detected via flow cytometry, and that infiltrating T_H_1 cells play a minor role with anti-tumor immunity when compared to CTLs.

We recognize that our data do not determine whether anti-tumor T-cells during tumor progression are decreasing in abundance, repolarizing to either T_H_2 or T_H_17 cell responses, or both. Other studies suggest that strong inflammatory T_H_17 cell activity drives tumorigenesis and angiogenesis and interferes with type-1 T-cell activity [29, 30]. Our results can neither confirm nor refute these studies. Therefore, the interplay between T-cell subsets and other infiltrating immune cells needs to be further explored. We recognize that four stage IV tumors are not enough to draw concrete conclusions; therefore we combined this group with stage III tumors when analyzing immune activity in advanced stage disease. Further analysis involving these stage IV tumors was limited to determining trends within the whole tumor population collected. We recognize that heterogeneity exists within each tissue collected; therefore we obtained samples from multiple sites to represent each specimen as a whole and to mitigate the chance of randomly selecting a unique compartment within each tissue that may be misrepresentative. However we do not know whether the assembly of samples obtained truly represented each specimen. That acknowledged, by culturing relatively large, minced tumor specimens, we increased the possibility of more broadly sampling the TME than can be achieved by tissue microarrays.

Our study champions a growing movement within the gastrointestinal cancer research community to incorporate an ‘immunoscore’ with current TNM staging [27]. The originators of this novel concept suggest that T-cell activity can be evaluated through IHC. Our study supports this concept, and reinforces the efficacy of this practice by measuring type-1 gene activity and chemokine secretion. This novel avenue for evaluating immune activity in a tumor will also provide practitioners with better insights into patients' odds for survival, which in turn may suggest which treatment strategy, if any, will be most appropriate. By surveying secreted immune mediators within the context of CRC, we underpin the basic perception of immune cell infiltration into solid tumors. This method will open avenues for better understanding disease progression and improve prognosis for many diseases.

This novel method could also be a way of testing pharmacological interventions in clinical specimens. Only a small group of CRC patients will derive a clinical benefit from each individual treatment, while all patients are at risk for toxicity when administered these drugs [31]. This has initiated a growing argument that some chemotherapeutic agents may adversely affect the immunological infiltrate in solid tumors. We can learn much more about the efficacy of chemotherapeutic interventions by culturing tumors that are infiltrated by immunosuppressive cells [32]. In mice, 5-flurouracil has been shown to inhibit myeloid-derived suppressor cells (MDSCs) allowing for an increase in T-cell activity [33]. Recently shown in a CRC mouse model, irinotecan increases immunosuppression by inhibiting the effect 5-flurouracil has on MDSCs [34]. Therefore, the idea of evaluating the effect of chemotherapies in our novel culturing method is intriguing, where it could be extended to predicting which patients will respond to chemotherapies. Developing new chemotherapeutic agents may not be the only specific way to benefit the patient population. Additionally we need a better understanding of current therapies so that patients may be treated more appropriately. Our novel method could be a tool for testing the impact of therapeutic agents on the immunological milieu, and determine which therapy is most likely to benefit the patient.

## MATERIALS AND METHODS

### Patient enrollment

Forty-nine patients were enrollment after providing informed consent. A HIPAA-approved record release authorization was obtained to grant access to each patient's protected health information. All potential patients required an initial diagnosis of any stage CRC prior to surgery. Forty-four separate tumor specimens were obtained in conjunction with the Department of Pathology at BUMC (Supplemental Table 2). This study received IRB approval, and includes protocol number, IRB#011-030. Full TNM staging was performed by a trained pathologist. The gender and age of each patient, as well of clinicopathological features, were recorded for later analysis. The identity of each patient remained blinded from the study's investigators. Due to the small size of the study, a larger cohort of patients was obtained from TCGA (http://cancergenome.nih.gov/) and utilized for validating results pertaining to clinicopathological features when appropriate [28].

### Tissue procurement

Tissues were excised from the patients and immediately delivered to the Department of Pathology and thoroughly examined by trained staff pathologists. 0.5-1.0 grams (approximately dime-quarter size) of each specimen were obtained by staff pathologists who were all instructed to collect multiple samples from at least three cross-sections of each tumor and to avoid sampling from the tumor surface and obvious areas of necrosis and ulceration. In total, approximately 4-6 samples were collected from each tissue. Masses initially diagnosed as adenomatous polyps were not normally collected. Normal adjacent mucosa was collected no more than 5 cm from the tumor site. Tissues were stored on ice or at 4°C in a covered sterile Petri dish contained in a protective secondary container, delivered to the GI Cancer Research Laboratory, and processed no more than 30 minutes after the completion of surgery. At no time were tissues frozen or placed in any fixative before analysis. Multiple portions of each tissue were preserved in RNA*later*^®^ (Qiagen, Valencia, CA) per manufacture's specifications and stored at −80°C for later RNA extraction.

### Ex-vivo tissue cultures and multiplex cytokine analysis (immunoassay)

Tissues extracted from the colon or rectum were removed from fatty tissues using a sterile No. 11 surgical blade, and washed with 1.0 mM DTT (Sigma, St Louis, MO) in Hank's Balanced Salt Solution (HBSS) without calcium, magnesium or phenol red (Life Technologies, Carlsbad, CA) for 15 minutes at 4°C on a rotator plate to remove the mucous layer, and rinsed three more times with plain ice cold HBSS to remove the DTT. To ensure that tissues had equal amounts of surface area during *ex-vivo* culturing, precisely 0.20 grams of CRCs and normal adjacent mucosae were minced (exactly 80 cuts) into smaller pieces (<1mm^3^) using a sterile No. 11 surgical blade. Minced tissue pieces were then fully submerged in 4 mL of Iscove's Modified Dulbecco's Medium (IMDM) (Life Technologies) plus 10% fetal calf serum (FCS) (Life Technologies), and 1X penicillin, streptomycin (Life Technologies), 1X gentamicin and amphotericin (Life Technologies) in vertically standing 25 cm^2^ tissue culture flasks, and incubated at 37°C in 5% CO_2_. Supernatants were collected after 16 hours and stored at −80°C, then thawed and centrifuged at 1500 rpm for 5 minutes to clear away debris, and assayed for secreted CCL2, CCL3, CCL4, CCL5, CCL11, CX3CL1, CXCL1, CXCL10, IFN-γ, IL-4, IL-5, and IL-13 using the EMD Millipore's MILLIPLEX *MAP* Human Cytokine/Chemokine Luminex kit system (Merck KGaA, Darmstadt, Germany). Data were analyzed using Bio-Plex Manager 6.0 (Bio-Rad, Hercules, CA). Eleven tumors and 20 normal mucosae samples were inadequate for immunoassay multiplexing.

### Flow cytometric analysis of infiltrating T-cells

Approximately 0.5 grams of each excised tumor (n=16) and normal adjacent tissue (n=15) were disaggregated into single-cell suspensions using 2.5 mg/mL collagenase D (Roche, Basel, Switzerland), 1 mg/mL hyaluronidase (Sigma), and 20 units/mL DNase I (Sigma) in phosphate-buffered saline (PBS) (Life Technologies) and incubated for 2 hours at 37°C with mild agitation and vortexing every 15 minutes. Suspensions were passed through 70 μM nylon cell strainers (BD Biosciences, San Jose, CA) to remove aggregates. Cells were stimulated with 50 ng/mL phorbol 12-myristate 13-acetate (PMA) and 1 μg/mL ionomycin and treated with GolgiPlug (BD Biosciences) for 12 hours at 37°C in 5% CO_2_. Cells were stained with LIVE/DEAD^®^ Fixable Aqua Dead Cell Stain (Life Technologies) and permeabilized using BD Cytofix/Cytoperm^TM^ Plus Fixation/Permeabilization Kit, then stained for PE-Cy^TM^7-CD45 (clone HI30), APC-Cy^TM^7-CD3 (clone SK7), Pacific Blue^TM^-CD4 (clone RPA-T4), PerCP-Cy^TM^5.5-CD8 (clone RPA-T8), PE-IFN-γ (clone 4S.B3) (BD Biosciences), and APC-Granzyme B (clone GB12) (Invitrogen, Carlsbad, CA). Analysis was performed using a FACSCanto II cytometer (BD Biosciences) and data were analyzed using FlowJo software. Between 10,000 and 50,000 live cells were collected from each tissue. All antibodies were tested against isotype controls (clone MOPC-21) (BD Biosciences). Markers were gated similarly between each tumor and corresponding normal mucosa using the tumor's isotype control stained lymphocyte population.

### Real-time PCR analysis of immune biomarkers

RNA was extracted and genomic DNA was removed using RNeasy^®^ Plus Mini Kit (Qiagen). RNA was converted to cDNA using the Advantage RT-for-PCR Kit (Clonetech Laboratories, Mountain View, CA) following manufacture's specifications. One tumor yielded inadequate RNA, and twenty-two normal mucosae were not collected from surgically resected specimens. Gene expression was analyzed using quantitative real-time QuantiTect SYBR Green PCR kit (Qiagen) and the StepOnePlus Real-Time PCR System (Applied Biosystems, Carlsbad, CA) following manufacture's specifications. Primer sequences were obtained from RTPrimerDB (http://www.rtprimerdb.org/index.php), the PrimerBank database (http://pga.mgh.harvard.edu/primerbank/) and Quantitative PCR Primer Database (QDDP) (http://www.rt-pcr.info/qppd-quantitative-pcr-primer-database/) (Supplemental Table 3), and were evaluated *in silico* using the UCSC Genome Browser (http://genome.ucsc.edu/) to ensure that coding sequences straddled multiple exons and that binding sites avoided single-nucleotide polymorphisms [35-38]. Primers were purchased from Integrated DNA Technologies (Coralville, IA). *GAPDH* was used as an internal normalizing control.

### Statistical analysis

Graphpad Prism 6.0 software (Graphpad Software, San Diego, CA) was used to generate all correlation coefficients (*r*) and *P* values. Transcriptional data, cytokine secretion, T-cell frequency, and all other data were analyzed using Student's *t*-test. One-way ANOVA test or chi-square tests were used when determining linear trends. Contingency tables were analyzed using Fisher's exact test. The R software package “Hierarchical Clustering” version 1.1.23-r7 (http://www.wessa.net/) consisted of Ward's method [39].

## SUPPLEMENTARY MATERIAL, FIGURES


