# Supplementary material for: Active secretion of CXCL10 and CCL5 from colorectal cancer microenvironments associates with GranzymeB+ CD8+ T-cell infiltration
Source: Oncotarget. 2014 Dec 11;6(5):2981–91. doi: 10.18632/oncotarget.3205 (PMC4413778; doi:10.18632/oncotarget.3205)
Supplement: Supplementary file 1 [file oncotarget-06-2981-s001.pdf]

Active secretion of CXCL10 and CCL5 from colorectal cancer microenvironments associates with GranzymeB^{+} CD8^{+} T-cell infiltration

Supplementary Material

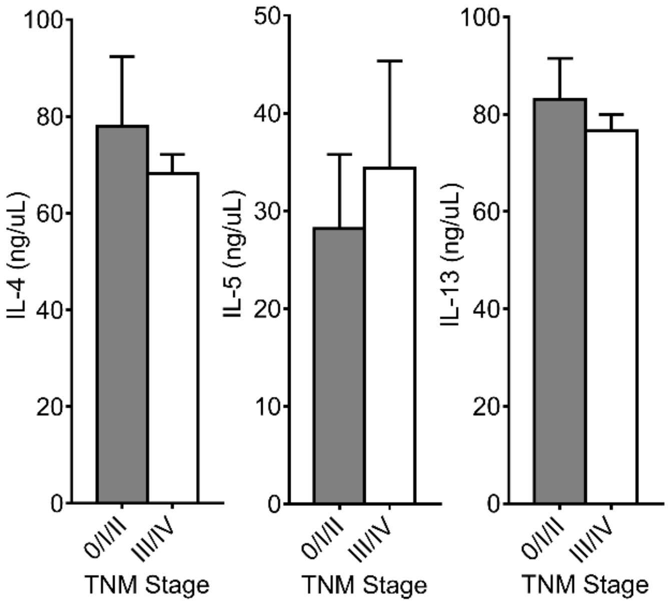

Suppl. Fig. 1: *Secretion of Type-2 T-cell chemokines from CRCs.*

Concentrations of secreted IL-4, IL-5, and IL-13 were measured from recently resected tissues minced into <1mm^3 pieces and placed into culture media for 16 hours. Millipore's MILLIPLEX *MAP* Human Cytokine/Chemokine Luminex kit system was used to quantify chemokines. Stage 0/I/II n=18 and stage III/IV n=15.

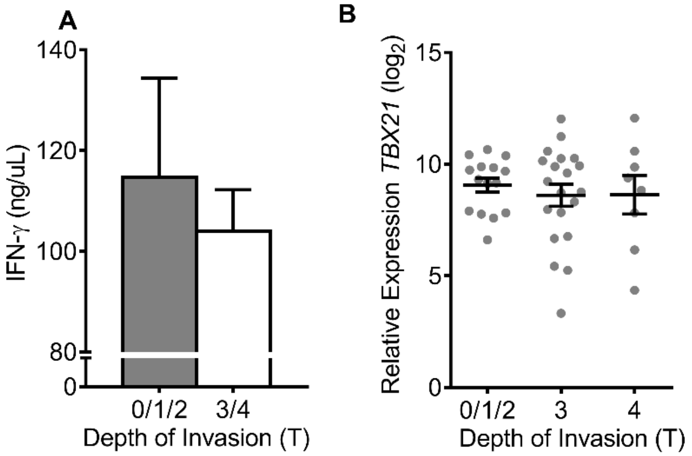

Suppl. Fig. 2: *Secretion of IFN-γ and expression of* TBX21 *among depth of invasion of CRCs.* A, IFN-γ secretion were measured from recently resected tissues minced into <1mm^3 pieces and placed into culture media for 16 hours, and B, $\log_2$ transformed *TBX21* expression among degree of invasion depth (T). IFN-γ was quantified using Millipore's MILLIPLEX *MAP* Human Cytokine/Chemokine Luminex kit system. *TBX21* expression was measured via real-time PCR using QuantiTect SYBR Green PCR kit (Qiagen). T0/1/2 n=15, T3 n=20, and T4 n=8.

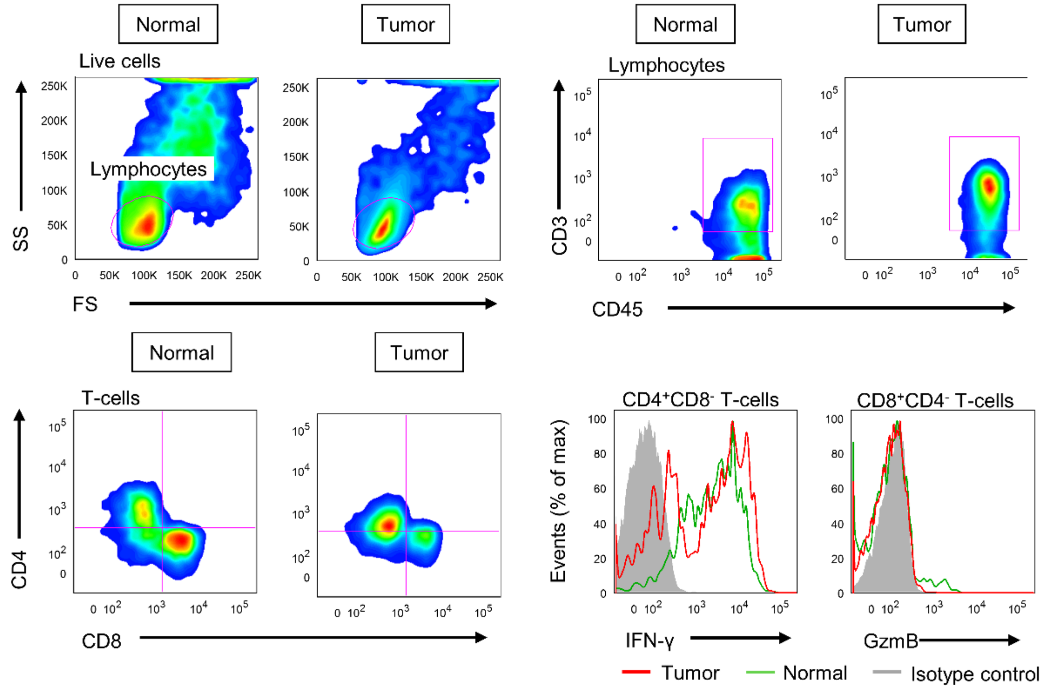

Suppl. Fig. 3: *Gating strategy used to identify T-cells subsets from one stage IV 'Lo' CRC.*

Recently resected normal mucosa and tumor tissue specimens were disaggregated into single cell suspensions, stimulated with PMA and ionomycin, treated with GolgiPlug, and then stained for seven-color flow cytometric analysis.

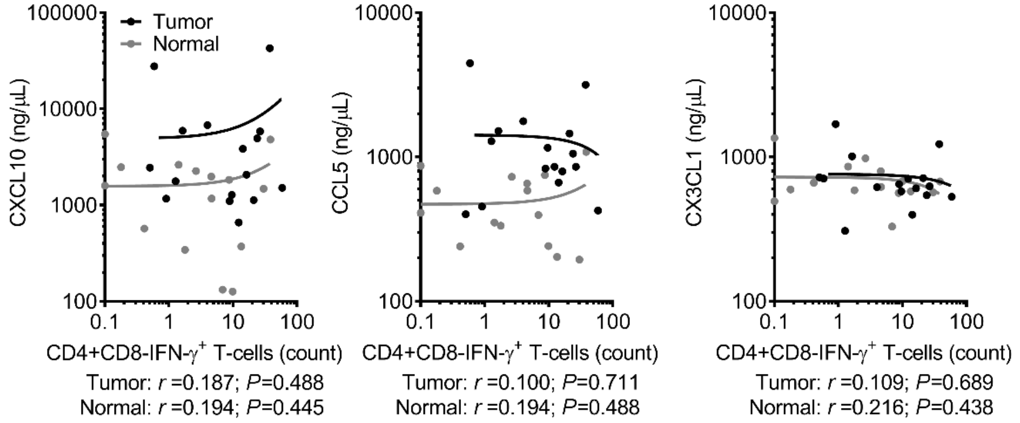

Suppl. Fig. 4: Dot plots and correlation coefficients (r) of chemokine concentrations from recently resected tissues minced into <1mm^3 pieces and placed into culture media for 16 hours versus frequency of IFN-γ$^+$ T$_H$1 cells determined by flow cytometry. Millipore's MILLIPLEX *MAP* Human Cytokine/Chemokine Luminex kit system was used to quantify chemokines. Normal n=15 and tumor n=16.

Suppl. Table 1: Average relative expression of genes involved in T-cell chemotaxis and T_H1 immunity. 'Hi' (n=20) or 'Lo' (n=23). All genes were normalized to *GAPDH* and Student's *t*-test was performed to determine significance.

Average relative expression of genes involved in T-cell chemotaxis and T_H1 immunity

Gene	Hi	Lo	P value
CXCL9	431.41	65.49	<0.001
CXCL10	423.42	53.24	<0.001
CXCL11	529.51	82.47	0.001
CCL2	48.24	16.49	0.012
CCL3	41.50	25.26	0.105
CCL4	50.56	18.20	<0.001
CCL5	85.09	19.35	<0.001
CCL11	197.69	62.44	0.018
CX3CL1	10.08	5.96	0.034
IFNG	141.41	54.17	0.040
TBX21	1485.50	263.51	<0.001
IRF1	9.45	4.67	<0.001
GNLY	148.95	53.60	0.005
GRZB	181.37	58.19	0.031
ICAM1	92.84	56.00	0.133

Suppl. Table 2: Forty-nine patients were enrollment after providing informed consent and signing a HIPAA-approved record release authorization. Forty-four separate tumor specimens were obtained from the Department of Pathology at BUMC. Full TNM staging was performed by a trained pathologist. The gender and age of each patient, as well as the primary tumor site were recorded for further clinicopathological analysis. Samples types that were collected and analyzed for each CRC are listed here.

Clinicopathological characteristics and samples types collected for each CRC

		Tissue Culture		RNA		Flow Cytometry		Primary			
Patient #	Date collected	Normal	Tumor	Normal	Tumor	Normal	Tumor	Tumor	Stage	Age	Sex
1	8-Mar-11	x	x	x	x	x	x	x	x	65	F
2	25-May-11	x	x	x	Y	x	x	Rectum	IVB	57	F
3	2-Jun-11	x	x	x	Y	x	x	Colon	IIA	62	M
4	9-Jun-11	x	x	x	Y	x	x	Rectum	I	45	M
5	15-Jun-11	x	x	x	Y	x	x	Rectum	IIA	52	F
6	22-Jun-11	x	x	x	Y	x	x	Rectum	IIA	40	M
7	22-Jun-11	x	x	x	Y	x	x	Colon	IIA	61	M
8	24-Aug-11	x	x	x	Y	x	x	Colon	I	59	M
9	7-Sep-11	x	Y	x	Y	x	x	Colon	I	69	M
10	Not Collected	x	x	x	x	x	x	x	x	65	M
11	31-Jan-12	x	Y	x	Y	x	x	Colon	IIIC	33	M
12	3-Feb-12	x	Y	x	Y	x	x	Rectum	IIIA	82	M
13	Not Collected	x	x	x	x	x	x	x	x	46	F
14	15-Feb-12	x	Y	x	Y	x	x	Rectum	I	51	M
15	1-Mar-12	x	x	x	Y	x	x	Colon	I	55	M
16	2-Mar-12	x	Y	x	Y	x	x	Rectum	IIA	61	M
17	12-Mar-12	x	Y	x	Y	x	x	Colon	IIA	50	M
18	28-Mar-12	x	x	x	Y	x	x	x	IIIB	52	F
19	29-Mar-12	x	Y	x	x	x	x	Colon	IIIB	59	M
20	29-Mar-12	x	Y	x	Y	x	x	Colon	IIIA	57	F
21	4-Apr-12	x	Y	x	Y	x	x	Colon	IIIB	46	F
22	9-Apr-13	x	Y	x	Y	x	x	Colon	I	49	M
23	23-Apr-12	x	x	x	Y	x	x	Rectum	IIIB	63	M
24	17-May-12	Y	Y	x	Y	x	x	Rectum	I	79	M
25	25-May-12	Y	Y	Y	Y	x	x	Rectum	IIIB	78	M
26	30-May-12	Y	Y	Y	Y	x	x	Rectum	IIB	72	M
27a	6-Jun-12	Y	Y	Y	Y	x	x	Rectum	IIIC	65	F
27b	6-Jun-12	Y	Y	Y	Y	x	x	Rectum	IIIC	65	F
28	Not Collected	x	x	x	x	x	x	x	x	49	M
29	22-Aug-12	Y	Y	Y	Y	x	x	Colon	IIIB	88	F
30	Not Collected	x	x	x	x	x	x	x	x	61	F
31	20-Sep-12	Y	Y	Y	Y	Y	Y	Colon	IVB	66	M
32	8-Oct-12	Y	Y	Y	Y	Y	Y	Rectum	I	61	F
33	11-Oct-12	Y	Y	Y	Y	Y	Y	Colon	IVA	31	M
34	7-Dec-12	Y	Y	Y	Y	Y	Y	Rectum	I	51	F
35	17-Jan-13	Y	Y	Y	Y	Y	Y	Colon	IIA	63	F
36	6-Feb-13	Y	Y	Y	Y	Y	Y	Colon	0	64	M
37	4-Mar-13	Y	Y	Y	Y	Y	Y	Rectum	I	90	F
38	10-Mar-13	Y	Y	Y	Y	Y	Y	Colon	IV	58	M
39	24-Apr-13	Y	Y	Y	Y	Y	Y	Colon	IIIC	79	F
40	24-Apr-13	Y	Y	Y	Y	Y	Y	Rectum	IIIA	61	M
41a	1-May-13	Y	Y	Y	Y	x	x	Colon	IIA	74	M
41b	1-May-13	Y	Y	Y	Y	Y	Y	Colon	IIA	74	M
42	1-Jul-13	Y	Y	x	Y	x	Y	Colon	IIA	66	F

43	16-Aug-13	Y	Y	Y	Y	Y	Y	Colon	IIA	55	M
44	21-Aug-13	Y	Y	Y	Y	Y	Y	Rectum	IIA	45	M
45	3-Sep-13	Y	Y	Y	Y	Y	Y	Colon	IIA	84	M
46	19-Sep-13	Y	Y	Y	Y	Y	Y	Colon	IIA	82	M
47	16-Oct-13	Y	x	Y	Y	x	x	Rectum	I	50	F
48	Not Collected	x	x	x	x	x	x	x	x	68	F
49	Not Collected	x	x	x	x	x	x	x	x	53	F

Suppl. Table 3: Forward (F) and reverse (R) primer sequences for real-time PCR analysis of genes of interest were obtained from online databases and validated *in silico* using the UCSC Genome Browser.

Primer sequences for real-time PCR

Gene	Sequence (5′ – 3′)	Size (bp)	Database ID
CCL2	F-GATCTCAGTGCAGAGGCTCG (20 bp)<br>R-TGCTTGTCCAGGTGGTCCAT (20 bp)	153	RTPrimerDB<br>1642
CCL3	AGTTCTCTGCATCACTTGCTG (21 bp)<br>CGGCTTCGCTTGGTTAGGAA (20 bp)	151	PrimerBank<br>4506843A1
CCL4	CGCCTGCTGCTTTTCTTACAC (21 bp)<br>CAGACTTGCTTGCTTCTTTTGG (22 bp)	126	RTPrimerDB<br>3535
CCL5	F-TACCATGAAGGTCTCCGC (18 bp)<br>R-GACAAAGACGACTGCTGG (18 bp)	199	RTPrimerDB<br>1650
CCL11	F-ATACCCCTTCAGCGACTAGAG (21 bp)<br>R-GCTTTGGAGTTGGAGATTTTTGG (23 bp)	169	PrimerBank<br>4506827A1
CD8A	F-ATGGCCTTACCAGTGACCG (19 bp)<br>R-AGGTTCCAGGTCCGATCCAG (20 bp)	104	PrimerBank<br>225007533C1
CXCL9	F-GACCTTAAACAATTTGCCCCAAG (23 bp)<br>R-CACATCTGCTGAATCTGGGTTTA (23 bp)	105	QPPD<br>3055
CXCL10	F-TTCAAGGAGTACCTCTCTCTAG (22 bp)<br>R-CTGGATTCAGACATCTCTTCTC (22 bp)	177	RTPrimerDB<br>3537
CXCL11	F-GACGCTGTCTTTGCATAGGC (20 bp)<br>R-GGATTTAGGCATCGTTGTCCTTT (23 bp)	148	PrimerBank<br>4885589A2
CX3CL1	F-ACCACGGTGTGACGAAATG (19 bp)<br>R-CTCCAAGATGATTGCGCGTTT (21 bp)	122	PrimerBank<br>4506857A1
GAPDH	F-TGAACGGGAAGCTCACTGG (19 bp)<br>R-TCCACCACCCTGTTGCTGTA (20 bp)	307	QPPD<br>96
GNLY	F-CCTGTCTGACGATAGTCCAAAAA (23 bp)<br>R-GACCTCCCCGTCCTACACA (19 bp)	100	PrimerBank<br>7108346A1
GZMB	F-TACCATTGAGTTGTGCGTGGG (21 bp)<br>R-GCCATTGTTTCGTCCATAGGAGA (23 bp)	124	PrimerBank<br>221625527C2
ICAM1	F-CTGCAGACAGTGACCATC (18 bp)<br>R-GTCCAGTTTCCCGGACAA (18 bp)	320	RTPrimerDB<br>3020
IFNG	F-CTCTTGGCTGTTACTGCCAGG (21 bp)<br>R-CTCCACACTCTTTTGGATGCT (21 bp)	230	PrimerBank<br>10835171A1
IRF1	F-CTCTGAAGCTACAACAGATGAG (22 bp)<br>R-GTAGACTCAGCCCAATATCCC (21 bp)	215	RTPrimerDB<br>3571
TBX21	F-CAGAATGCCGAGATTACTCAG (21 bp)<br>R-GGTTGGGTAGGAGAGGAGAG (20 bp	169	RTPrimerDB<br>7752
